# Screening of 44 Baltic Sea Cyanobacterial Strains for Antibacterial and Quorum Sensing Inhibitory Potential: Selection of Promising Candidates

**DOI:** 10.3390/antibiotics15040371

**Published:** 2026-04-03

**Authors:** Anna Toruńska-Sitarz, Robert Konkel, Agnieszka Ogrodnicka, Hanna Mazur-Marzec, Magdalena Socha, Donata Overlingė

**Affiliations:** 1 Department of Marine Biology and Biotechnology, Faculty of Oceanography and Geography, University of Gdańsk, Marszałka Piłsudskiego 46, 81-378 Gdynia, Poland; robert.konkel@ug.edu.pl (R.K.); a.ogrodnicka@gmail.com (A.O.); hanna.mazur-marzec@ug.edu.pl (H.M.-M.); magdalenasocha793@gmail.com (M.S.); 2 Marine Research Institute, Klaipeda University, Universiteto Av. 17, LT-92294 Klaipeda, Lithuania

**Keywords:** Baltic Sea, cyanobacteria, antibacterial activity, quorum quenching, bioactivity-guided fractionation

## Abstract

**Background/Objectives:** Cyanobacteria represent a diverse group of microorganisms capable of synthesizing a broad array of biologically active metabolites. Some of these compounds, believed to contribute to the ecological and evolutionary success of cyanobacteria, are increasingly being investigated for potential biomedical and biotechnological applications. They also hold promise in combating the growing threat of antimicrobial resistance (AMR). This screening study aimed to identify Baltic cyanobacterial strains with the potential to produce antibacterial compounds active against streptococci and mycobacteria, as well as quorum sensing inhibitors. **Methods/Results:** Extracts from forty-four cyanobacterial strains were tested using a broth microdilution assay. The most pronounced activity was observed for extracts derived from two Pseudanabaenaceae strains (KUCC C3 and C4), two *Anabaena* spp. strains (CCNP 1405 and CCNP 1406), and *Aphanizomenon* sp. KUCC C1. Inhibition of quorum sensing was the most frequently detected activity, with 30% of the tested extracts inhibiting violacein production in *Chromobacterium violaceum* ATCC 12472. Growth inhibition of Gram-positive bacteria was less common: 16% of cyanobacterial strains inhibited *Streptococcus pyogenes* ATCC 12344, and 11% inhibited *Mycobacterium smegmatis* ATCC 14468. Bioassay-guided fractionation of *Aphanizomenon* sp. KUCC C1, followed by LC–MS/MS analysis, revealed the presence of glycerolipids and glycolipids, including diacylglycerols (DAGs) and galactosyldiacylglycerols (MGDGs and DGDGs), as major constituents of fractions exhibiting quorum quenching activity. **Conclusions:** These findings highlight the potential of Baltic cyanobacteria as a source of natural compounds capable of disrupting bacterial communication and growth, offering prospects for the development of novel antimicrobial and anti-virulence agents.

## 1. Introduction

The long evolutionary history of cyanobacteria is reflected in their extensive adaptations that enable the colonization of diverse environments, including the biosynthesis of a broad spectrum of bioactive compounds, predominantly secondary metabolites. These metabolites are not directly involved in primary metabolism but confer adaptive advantages under biotic and abiotic stress. They mediate defense against environmental stressors and grazers, modulate interactions with co-occurring organisms through allelopathic mechanisms, and contribute to chemical communication within the microbial loop [1]. To date, the mechanisms and underlying causes governing the production of specific secondary metabolites remain poorly understood. The ecological significance of secondary metabolite production has been investigated most extensively, particularly with respect to microcystins (MCs), which are among the most frequently detected and best-characterized toxins produced by cyanobacteria [2]. Intracellular microcystins are involved in carbon and nitrogen metabolism and contribute to antioxidant defense, whereas extracellular MCs participate in cell-to-cell communication and colony formation [3].

In natural environments, cyanobacterial cells and colonies are commonly associated with heterotrophic bacteria residing on their surfaces or within their mucilaginous sheaths, collectively forming a distinct microenvironment, cyanosphere [4]. Within this microhabitat, interactions among microorganisms are highly diverse. Mutualistic relationships contribute to niche formation and community stability. These include carbon provision via cyanobacterial exopolysaccharides that support heterotrophic growth, reciprocal exchanges of vitamins and other metabolites, and complementary sulfur cycling pathways [5,6,7]. These interactions may also shift toward competitive and antagonistic dynamics, potentially involving the production of antibacterial compounds that influence the structure of the associated microbiome, e.g., Refs. [8,9]. This naturally occurring phenomenon has recently attracted increasing attention in the context of practical applications and is being actively explored in vitro.

Primarily investigated in basic research, antibacterial effects rank as the third most frequently described bioactivity of cyanobacteria, following cytotoxicity/lethality and enzyme inhibition, and account for approximately 10% of all documented biological activities, according to recent reviews [10,11]. Baltic cyanobacteria remain comparatively underexplored in this context [12]. Since the second half of the 20th century, Baltic cyanobacteria have attracted considerable scientific attention mainly due to their large-scale, toxic summer blooms and the associated environmental impacts [13]. The earliest available report indicating the antibacterial potential of Baltic cyanobacteria was published in 1999 [14]. In that study, an extract obtained from the *Nodularia spumigena* strain did not exhibit antibacterial activity. In contrast, aqueous and hexane extracts derived from field-collected cyanobacterial bloom material dominated by *Microcystis*, *Anabaena*, and *Nodularia* genera inhibited the growth of *Bacillus subtilis* and *Staphylococcus aureus* (2 mg per disk; agar diffusion assay). In our previous study [15], we evaluated the antibacterial activity of methanol extracts obtained from nine phytoplankton samples collected in the Curonian Lagoon. The contribution of cyanobacteria in these phytoplankton communities accounted for 3–43% of the total biomass. The extracts inhibited the growth of the environmental strains *Aeromonas salmonicida* and *Vibrio diazotrophicus*, as well as *Enterococcus faecium* and a clinical isolate of methicillin-resistant *Staphylococcus aureus* (MRSA), by 50% in a broth microdilution assay at concentrations ranging from 125 to 500 µg/mL. Similar findings were reported by Mazur-Marzec et al. [16], who demonstrated that 9 (33%) out of 27 Baltic cyanobacterial strains exhibited antimicrobial activity against at least 1 of the 22 tested bacterial isolates. The ethanol extract of *Phormidium* sp. CCNP1317 demonstrated the broadest and strongest activity, inhibiting six fouling Gammaproteobacteria with MIC_50_ values ranging from 0.6 to 4 µg/mL. Extracts of *Microcystis aeruginosa* CCNP1102 were also active against both Gram-positive and Gram-negative bacteria, including *Staphylococcus aureus*, *Micrococcus luteus*, *Serratia marcescens*, and *Pseudomonas aeruginosa* (MIC_50_: 42–168 µg/mL).

Despite the development of alternative approaches such as targeted delivery systems, bacteriophages, and physicochemical methods, natural products and their derivatives still constitute the majority of approved small-molecule antibiotics [17,18]. Screening natural extracts for antibacterial activity remains a fundamental strategy in new drug discovery, particularly in the context of rising antimicrobial resistance (AMR). The enormous chemical diversity of microorganisms continues to represent a highly promising source of novel antibacterial agents [19]. Compared with compounds derived from terrestrial microbes, marine metabolites exhibit distinctive chemical features, including unusual ring systems, halogenation, and nonstandard branching. These characteristics may give rise to divergent and, in some cases, entirely novel antibacterial mechanisms of action, as well as potentially reduced susceptibility to cross-resistance relative to conventional antibiotics [20]. The aim of our study was to screen Baltic cyanobacterial strains for the presence of quorum sensing inhibitors (i.e., quorum quenching compounds) and antibacterial compounds active against streptococci and mycobacteria, addressing the limited number of studies investigating these activities in Baltic cyanobacteria. Based on the screening results, one of the most active strains, *Aphanizomenon* sp. KUCC C1, was selected for further investigation using bioassay-guided fractionation to identify bioactive fractions and assess their quorum quenching potential.

## 2. Results

### 2.1. Antibacterial Activity of Crude Extracts

Out of the 44 tested extracts, 7 inhibited the growth of *S. pyogenes* ATCC 12344 and 5 inhibited the growth of *M. smegmatis* ATCC 14468 (Table 1 and Tables S1–S3). The crude extracts originated from cyanobacteria belonging to the orders Chroococcales (1 strain: *Microcystis* sp. CCNP 1106), Synechococcales (2 strains from the family Pseudanabaenaceae: KUCC C3 and KUCC C4), and Nostocales (5 strains: 4 *Anabaena* spp.—CCNP 1405, CCNP 1406, CCNP 1407, CCNP 1416, and *Aphanizomenon* sp. KUCC C1), with MIC (Minimal Inhibitory Concentration) values ranging between 32 and 1000 μg/mL. The lowest MIC value in the assay with *M. smegmatis* ATCC 14468 (32 μg/mL) was recorded for KUCC C3 and KUCC C4 (Table 1 and Table S3). These two cyanobacterial strains also inhibited *S. pyogenes* ATCC 12344, with MIC values of 250 μg/mL (Table 1 and Table S2). Extracts obtained from *Anabaena* spp. CCNP 1405 and CCNP 1406 showed the same low MIC value (250 μg/mL) in the assay with the tested *S. pyogenes* strain. In addition to inhibitory effects, some extracts induced moderate growth stimulation (>100% of control), reaching up to ~150–156% for *S. pyogenes* ATCC 12344 and ~130–140% for *M. smegmatis* ATCC 14468, predominantly at higher concentrations (1000–125 μg/mL, Tables S1 and S2).

Thirteen extracts (~30%) inhibited quorum sensing (measured by violacein synthesis) in *C. violaceum* ATCC 12472, as evidenced by the absence of detectable violacein production (Table 1). Except for *Microcystis* sp. CCNP 1106, quorum sensing inhibitory activity coincided with antibacterial activity against the tested Gram-positive strains. The absence of visible violacein production was also observed for four Oscillatoriales strains belonging to the genus *Limnoraphis* (CCNP 1314, CCNP 1315, CCNP 1316, and CCNP 1324), *Microcystis aeruginosa* CCNP 1102 (Chroococcales), and *Nostoc* sp. CCNP 1438 (Nostocales). Violacein production was inhibited at concentrations as low as 63 μg/mL for *Aphanizomenon* sp. KUCC C1 and 32 μg/mL for *Anabaena* sp. CCNP 1406 (Table 1).

### 2.2. Antibacterial Activity–Guided Fractionation of the KUCC C1 Extract

**Figure 1:**
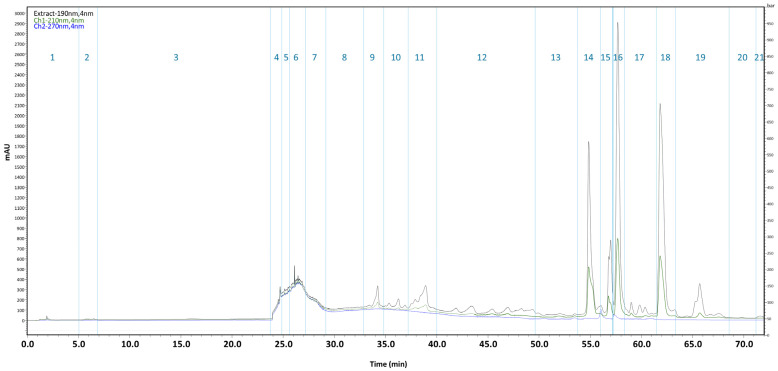
Flash chromatography chromatogram of the KUCC C1 crude extract recorded at three detection wavelengths: 190 nm (black), 210 nm (green), and 270 nm (blue). A total of 21 fractions were collected, as indicated by vertical lines and consecutive numbering.

A bioactivity-guided fractionation approach was applied in this study. In the first stage, 52 fractions were obtained from the KUCC C1 crude extract, of which 27 exhibited antibacterial activity (13 inhibited bacterial growth, whereas 14 inhibited only violacein production; Table S4). Fractions no. 24–35, eluted from the column with 80% and 100% methanol, were pooled. Based on the absorption intensities of different wavelengths in the chromatogram, they were subsequently separated into 21 subfractions (Figure 1). In the subsequent assay against *C. violaceum* ATCC 12472, fractions no. 8–14, 18–19, and 21 displayed activity, predominantly at concentrations above 1 mg/mL (Table 2 and Table S5). Fraction 14 selectively inhibited violacein production without affecting bacterial growth and was the most potent, with activity observed at 63 μg/mL. This effect was confirmed by colony formation on Mueller–Hinton agar from samples collected from the wells, with comparable CFU counts (~10^8^ CFU/mL), indicating a lack of bactericidal activity. In contrast, no growth was observed for samples exhibiting bactericidal effects (fractions 8–11 and 19 at >8 ng/mL, fraction 12 up to 1000 µg/mL, and fraction 18 up to 4000 µg/mL).

**Figure 2:**
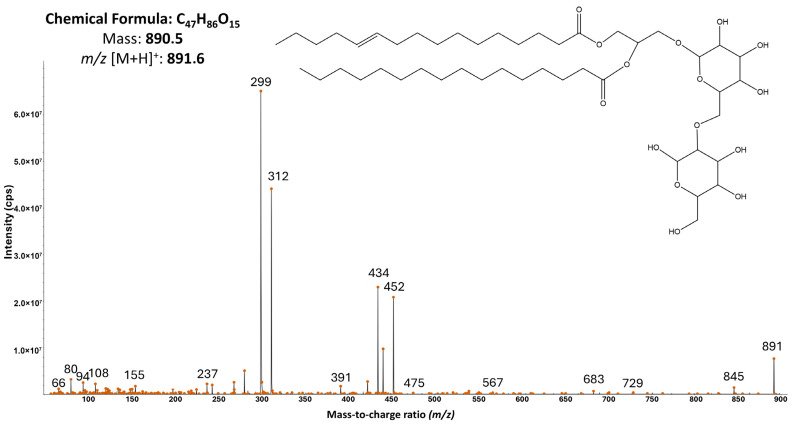
Probable chemical structure and MS/MS fragmentation spectrum of digalactosyldiacylglycerol (DGDG 16:1/16:0) with precursor ion [M + H]^+^ at *m*/*z* 891.6. The position and configuration of the double bond are tentative and cannot be unambiguously determined based solely on MS/MS analysis.

Analysis of the 21 fractions’ composition and comparison with entries in the CyanoMetDB database did not allow the identification of any previously described cyanobacterial metabolites. The fractions remained complex mixtures of compounds; however, for fractions 8–14, a putative class of metabolites could be proposed (Table S6). Interpretation of the MS/MS spectra suggested that these metabolites predominantly belong to glycerolipids and glycolipids, including diacylglycerols (DAGs) and galactosyldiacylglycerols (MGDGs and DGDGs), as well as their analogs. One of the most intense ions, observed at *m*/*z* [M + H]^+^ 891.6, was identified based on its diagnostic product ion spectrum as DGDG (16:1/16:0) (Figure 2). The spectrum displayed the expected DAG-core fragment series resulting from two consecutive glycosidic cleavages (Δm ≈ 162 Da), yielding fragment ions at *m*/*z* 729 and *m*/*z* 567. Additional fragments corresponding to neutral losses of the fatty acid 16:1 (Δm ≈ 255 Da) produced ions at approximately *m*/*z* 312. Furthermore, a fragment corresponding to the loss of the glycerol backbone (Δm ≈ 92 Da) was also observed, yielding an ion at *m*/*z* 475.

## 3. Discussion

Antibacterial activity of Baltic cyanobacteria has been reported in only a limited number of studies and has been evaluated against a narrow range of clinically relevant bacterial groups. To expand knowledge in this area, we investigated cyanobacteria isolated from the brackish waters of the Southern Baltic Sea. The study revealed several strains exhibiting antimycobacterial and antistreptococcal activities, as well as quorum quenching potential. These findings enabled the selection of candidates for more in-depth investigation and highlighted the largely unexplored potential of Baltic cyanobacteria. In addition to inhibitory effects, some extracts induced moderate growth stimulation, which may have both ecological and applied relevance. Cyanobacteria can stimulate the growth of selected bacteria depending on strain-specific metabolite profiles [21]. Conversely, such effects may be exploited in postbiotic production, as cyanobacteria-derived substrates can modulate microbial metabolism and enhance the bioactivity of extracellular products [22].

The target Gram-positive bacterial groups were selected due to their recent inclusion in the World Health Organization (WHO) priority pathogen list; specifically, rifampicin-resistant *Mycobacterium tuberculosis* as critical priority pathogens, and macrolide-resistant Group A streptococci as medium priority pathogens [23]. In this study, *Mycobacterium smegmatis* was used as a low-pathogenic, fast-growing model organism for early-stage anti-tuberculosis drug discovery [24]. Notably, previous research on the antibacterial potential of marine-derived extracts and isolated compounds has primarily focused on ESKAPE pathogens [25], with *Staphylococcus aureus* being the predominant Gram-positive target [10,25,26]. According to available bibliographic data, cyanobacteria appear to be a rich source of antibacterial compounds, particularly active against Gram-positive bacteria [10,26]. As early as the late 1970s, the first isolated and structurally characterized cyanobacterial antibiotic, malyngolide, was reported. This compound exhibited activities similar to those investigated in our study, including antibacterial effects against *S. pyogenes* and *M. smegmatis*, as well as quorum sensing inhibition in *C. violaceum* [27,28].

Among the Baltic cyanobacteria investigated in the present study, active extracts were obtained from representatives of the genera *Microcystis*, *Anabaena*, and *Aphanizomenon*, as well as from members of the family Pseudanabaenaceae. Of these, only *Anabaena* CCNP 1406 had previously demonstrated antibacterial activity against other Gram-positive bacteria, including non-resistant isolates of *S. aureus* and *Micrococcus luteus*. The reported MIC_50_ values ranged from 250 to 300 µg/mL [16], which are lower than those determined in the present study. In the same study [16], no antibacterial activity was detected for the other strains active in our current investigation. In the present work, anti-streptococcal and antimycobacterial activity was observed not only in *Anabaena* CCNP 1406 but also in *Anabaena* spp. CCNP 1405, 1407 and 1416, as well as in *Microcystis* sp. CCNP 1106. Members of *Anabaena* and *Microcystis* are among the cyanobacterial taxa with the highest number of reported antibacterial compounds [26]. Several metabolites produced by these microorganisms have been shown to inhibit Gram-positive bacteria, including bromoanaindolone, aeruginazole A and D, and kawaguchipeptins A and B [26]. Notably, antimycobacterial activity has been reported for microcystin-LR [29]. Cyanobacterial strains found to be active in the present study were not microcystin producers [16].

Thus far, no antibacterial metabolite derived from Baltic cyanobacteria has been reported. However, previous studies with the application of cyanobacteria bloom samples showed that compounds with such activity are produced by these microorganisms. For example, cyanobacteria from the Curonian Lagoon inhibited the growth of *S. aureus*, *E. faecium*, *A. salmonicida*, and *V. diazotrophicus* [15]. In the current work, the effects of Pseudanabaenaceae KUCC C3, C4, and *Aphanizomenon* sp. KUCC C1 extracts on *M. smegmatis* ATCC 14468 and *S. pyogenes* ATCC 12344 were studied for the first time. The results for strains from the family Pseudanabaenaceae are of particular interest. To the best of our knowledge, the antibacterial activity of these microorganisms against streptococci and mycobacteria has not been previously investigated. Notably, the extracts exhibited low MIC values (32 µg/mL). According to Sabotič et al. [25], extracts with MIC values below 100 µg/mL are considered promising and efforts to identify the active agent should be made. Data on the antibacterial activity of Pseudanabaenaceae species against other pathogens remain limited. Nevertheless, available studies suggest that members of this family generally exhibit activity against both Gram-positive and Gram-negative bacteria. For instance, two *Pseudanabaena* strains isolated from a lake in India inhibited bacterial growth with MIC values ranging from 125 to 500 µg/mL [2,30]. A sponge-associated *Pseudanabaena* cf. *persicina* from the North Aegean Sea demonstrated stronger activity against *S. aureus* (>10 mm inhibition zone) compared to Gram-negative bacteria (<10 mm) [31]. Similarly, *Pseudanabaena lonchoides* isolated from a freshwater stream in Turkey showed antibacterial effects against Gram-positive bacteria, with MIC values between 0.3 and 2.5 mg/mL [32]. In the study on antibacterial activity of *Limnothrix redekei*, fatty acids, including coriolic acid and 13-dimorphecolic acid, were indicated as responsible for inhibition of *S. aureus* growth [33]. Beyond direct metabolite-mediated activity, representatives of Pseudanabaenaceae may also contribute to antimicrobial applications through nanobiotechnology approaches. It has been demonstrated that extracts from strains belonging to this family can act as reducing and capping agents in the biosynthesis of silver nanoparticles, resulting in materials with confirmed bactericidal properties [34]. Although Pseudanabaenaceae strains KUCC C3 and KUCC C4 exhibited the strongest antibacterial activity (lowest MIC values), further analyses were not performed due to limited biomass availability. These strains are currently being cultivated and represent promising candidates for future studies.

In general, the potential of cyanobacterial metabolites as a source of lead structures for the development of novel antimycobacterial agents remains largely underexplored. This activity has been documented for both crude extracts (MIC 2.7–100 µg/mL) [35], (inhibition zones of 10–17 mm in a disk diffusion assay) [36] and isolated metabolites. Among the bioactive agents, ambiguine isonitriles, hapalindoles, hapalonamides, and eucapsitrione have been identified (MIC < 10 µM) [37,38,39]. Cyanobacteria were also reported to be active against *S. pyogenes*. The effects were recorded for extracts prepared with different solvents (acetone, methanol and water, MIC ≥ 50 mg/mL) [40]. The lipid/fatty-acid fractions usually showed higher activity (inhibition zones 16–20 mm) [41]. The anti-streptococcal activity was also proved for individual cyanobacterial metabolites, particularly hapalindoles, with MIC values ranging from 2 to 64 µg/mL, depending on the analog, and cybastacins, which display MIC_50_ values below 32 µg/mL ([26] and references therein).

In addition to direct antibacterial effects, we investigated the potential of the studied cyanobacteria to interfere with quorum sensing, a strategy targeting bacterial cell–cell communication mechanisms. This process, mediated by signaling molecules known as autoinducers, coordinates biofilm formation and the expression of virulence factors, without promoting the development of resistance [42]. In the future, such compounds could find application as standalone drugs or in combination with conventional antibiotics to enhance their efficacy. Quorum sensing reporter strains, such as *C. violaceum*, applied in our study, are typically used to detect interference with quorum sensing in screening assays. The activity was detected in one third of the samples tested, adding new data to the still limited number of studies reporting anti-QS activity in cyanobacterial extracts [8,43,44]. Here, we found that the representatives of Pseudanabaenaceae, *Aphanizomenon*, and *Anabaena* exhibited the highest activity (MIC < 250 µg/mL). In the only available published research on quorum quenching among listed taxa, Romero et al. [45] detected *Anabaena* sp. PCC 7120 metabolites that interfere with associated microbiota.

Among the strains exhibiting quorum sensing inhibitory activity, *Aphanizomenon* sp. KUCC C1 was selected for further analysis due to its activity at relatively low concentrations and the limited number of studies focusing on the biological activity of metabolites produced by this genus. The activity of the KUCC C1 crude extract, initially identified in a preliminary screening [46], was subsequently confirmed under replicated conditions in the present study. Thus far, few reports on the antibacterial activity of the *Aphanizomenon* genus have been published. Most cases concerned freshwater *Aphanizomenon flosaquae*, mainly due to the accessibility of its biomass in different forms, including commercial products [47], natural bloom material [41], and laboratory cultures [48].

Active fractions of KUCC C1 were found to be enriched in glycerolipids and glycolipids, particularly diacylglycerols and galactosyldiacylglycerols, suggesting that lipids which are abundant in cyanobacterial thylakoid membranes may contribute to the observed antibacterial and anti-QS effects [49]. Microalgal lipids belonging to MGDGs and DGDGs exhibit diverse biological activities, including bactericidal and antibiofilm effects ([50] and references therein). Although quorum sensing inhibition has not yet been reported for cyanobacterial MGDGs/DGDGs or purified compounds, structurally related glycerol-derived lipids, including alkylglycerols, exhibit anti-QS activity within the 20–795 μM range [51]. In the limited number of published studies, these compounds were shown to represent a promising tool in the fight against antimicrobial resistance (AMR) through alternative mechanisms. Monogalactosyldiacylglycerol containing a palmitoyl moiety (MGDG-palmitoyl), isolated from *Oscillatoria acuminata* NTAPC05, demonstrated bactericidal activity against ESBL-producing uropathogens, exceeding the efficacy of a fourth-generation cephalosporin, while remaining non-toxic to the HEK293 cell line [52]. Generally, antimicrobial activity of lipids produced by cyanobacteria is mainly attributed to the disruption of quorum sensing and biofilm formation, but also to membrane destabilization and inhibition of the electron transport chain in bacterial membranes was documented [53].

In conclusion, screening of 44 Baltic cyanobacterial strains identified three isolates exhibiting antibacterial or quorum quenching activity, which were selected as promising candidates for future studies, highlighting the potential of brackish water taxa as a source of bioactive compounds relevant to antimicrobial resistance (AMR). Representatives of the family Pseudanabaenaceae emerged as promising candidates for further investigation due to their activity against *S. pyogenes* and *M. smegmatis.* In parallel, further analysis of *Aphanizomenon* sp. KUCC C1 by bioassay-guided fractionation indicated potential involvement of glycerolipids and glycolipids in the observed quorum quenching activity. As related lipid standards are commercially accessible, targeted testing may facilitate verification of their contribution to the observed effects and elucidation of their mechanism of action, without prolonged isolation procedures.

Future studies will focus on the isolation of pure active compounds from three selected strains and on a range of additional assays, including testing against resistant clinical isolates, advanced quorum sensing inhibition models, and investigation of the underlying mechanisms of action.

## 4. Materials and Methods

### 4.1. Preparation of Cyanobacterial Extracts

Forty-four clonal, non-axenic strains from the phylum Cyanobacteriota were employed in the present study (Table S1). These microorganisms are deposited in the Culture Collection of Northern Poland (CCNP) at the Laboratory of Marine Biotechnology, University of Gdańsk. Cells were harvested in the exponential growth phase mainly by centrifugation (8 °C, 10 min, 3800 rpm; 5810 R, Eppendorf, Hamburg, Germany) or collected using a phytoplankton net (50 µm mesh size) and lyophilized. For each strain, 200 mg of dry biomass was extracted with 20 mL of 80% ethanol (Merck KGaA, Darmstadt, Germany). The extraction was performed twice by shaking for 15 min (Multi-Tube Vortexer, 2500 rpm, VWR, Radnor, PA, USA), followed by bath sonication (Sonorex, Bandelin, Berlin, Germany) and an additional 15 min of shaking. The combined extracts were centrifuged (10 min, 2500 rpm) and evaporated to dryness using a miVac Quattro concentrator (Genevac Ltd., Ipswich, UK). The extraction yield ranged from 6 to 61 mg per 200 mg of biomass. The dried extracts were stored at −20 °C. Just prior to antibacterial activity testing, the extracts were dissolved in sterile water containing 3% DMSO (Merck KGaA) and tested at concentrations ranging from 4 to 1000 µg/mL, in triplicate.

### 4.2. Extraction and Fractionation of KUCC C1 Biomass

Dry biomass of *Aphanizomenon* sp. KUCC C1 (11 g) was extracted twice with 500 mL 75% methanol. Each extraction step included 15 min of bath sonication followed by 30 min of shaking. The extracts were centrifuged (15 min, 4000 rpm, 4 °C), and the supernatants from both extractions were combined and diluted with Milli-Q water to approximately 10% methanol. The obtained extract was loaded onto Biotage^®^ Sfär C18 D column (Biotage, Uppsala, Sweden) and fractionated using a flash chromatography system (Shimadzu, Kyoto, Japan). The flow rate was set at 15 mL per minute. Elution was performed with increasing concentrations of methanol, and a total of 52 fractions were collected (Table S4).

After analysis (LC-MS/MS, Section 4.3) of fractions, one quarter of each fraction was evaporated and used for the bioassay on ATCC 12472 (Section 4.4, one repetition). The residual volume was evaporated to dryness in a separate vessel and fractions 24–35 were combined for preparative chromatography in a Jupiter^®^ Proteo 90 Å column (Phenomenex, Torrance, CA, USA) (based on the detected activity, Table S4). Briefly, the mobile phase consisted of 5% acetonitrile in Milli-Q water (phase A) and 100% acetonitrile (phase B), with a flow rate set to 12 mL/min. Gradient separation started at 5% of phase B and increased to 100% over 77 min. Based on the chromatogram (λ = 190, 210, and 270 nm; Figure 1), 21 fractions were collected. These fractions were subsequently analyzed using LC-MS/MS and evaluated in a broth microdilution assay against the ATCC 12472 strain (performed in duplicate, due to the limited quantity of the obtained fractions).

### 4.3. Mass Spectrometry

LC–MS/MS analyses were performed on a Sciex QTRAP 5500 tandem mass spectrometer (Sciex, Toronto, ON, Canada) coupled to an Agilent 1200 HPLC system (Agilent Technologies, Waldbronn, Germany). Aliquots of 5 µL were injected onto a Jupiter Proteo C12 column (150 × 4.6 mm, 4 µm, 90 Å; Phenomenex, Aschaffenburg, Germany) maintained at 40 °C. The mobile phases consisted of (A) Milli-Q water containing 5% acetonitrile (ACN) and (B) 100% ACN, both supplemented with 0.1% formic acid. The turbo ion spray source operated in positive ionization mode at 550 °C, with a spray voltage of +5.5 kV, and nebuliser and curtain gas pressures set to 60 psi and 20 psi, respectively. Data were acquired in information-dependent acquisition (IDA) mode. Ions within the *m*/*z* range of 500–1250 and with intensities exceeding 5 × 10^5^ cps were selected for fragmentation. The collision energy was set to 60 eV, and the dwell time was 10 ms. Raw data acquisition and processing were performed using Analyst 1.7.1 software (SCIEX, Toronto, ON, Canada, 2019). The acquired MS/MS spectra were searched against the CyanoMetDB database, version 3 (accessed on 30 October 2025) [54].

### 4.4. Antibacterial Assays

The broth microdilution assay was performed according to the guidelines of the European Committee on Antimicrobial Susceptibility Testing (EUCAST, http://www.eucast.org). The assay was conducted in 96-well polystyrene plates (96 Well EDGE Cell Culture Plates, NEST, Wuxi, China) following the procedure described in detail by Overlingė et al. [15]. Mueller–Hinton broth (Oxoid, Landsmeer, The Netherlands) was used as assay medium.

Three reference bacterial strains obtained from the American Type Culture Collection (ATCC) were used: *S. pyogenes* ATCC 12344, *M. smegmatis* ATCC 14468, and *C. violaceum* ATCC 12472. The starting inoculum in each experimental well was standardized to 5 × 10^5^ CFU/mL.

Two-fold serial dilutions of extracts/fractions were tested. For Gram-positive strains, bacterial growth after 36 h of incubation at 36 °C was assessed by measuring the optical density of each well at 620 nm using a SpectraMax^®^ i3 Platform (Molecular Devices, San Jose, CA, USA). The percentage of growth inhibition was calculated in comparison with the control (bacterial culture without the extract). MIC values, defined as the lowest concentration that completely inhibited bacterial growth, were also determined.

Plates inoculated with *C. violaceum* ATCC 12472 were assessed after 24 h of incubation at 36 °C for the presence or absence of purple pigment (violacein) (visual inspection and absorbance measurements at 585 nm). To distinguish between quorum sensing inhibition and bactericidal effect, samples from wells in which no pigment production was observed were plated onto Mueller–Hinton agar (100 µL). After 24 h of incubation, the presence or absence of bacterial growth was assessed. Lack of growth (no detectable CFU) was considered a bactericidal effect, whereas comparable CFU counts and colony formation relative to the control were interpreted as indicative of quorum sensing inhibition only.

For each assay, a solvent control (3% DMSO) was included and showed no effect on bacterial growth or violacein production. Sterility controls were also performed by replacing the bacterial inoculum with sterile saline solution. Positive controls included penicillin G (VWR Life Sciences, Leuven, Belgium) for *S. pyogenes* ATCC 12344 (MIC < 2 µg/mL) and rifampicin (Appli-Chem GmbH, Darmstadt, Germany) for *M. smegmatis* ATCC 14468 (MIC < 12 µg/mL). Vanillin (Warchem, Warsaw, Poland) was used as a positive control for quorum sensing inhibition in strain ATCC 12472 (<125 µg/mL).

## Figures and Tables

**Table 1:** Antibacterial activity of extracts from 44 cyanobacterial strains tested.

Extract Concentration [μg/mL]									
			1000	500	250	125	63	32	≤16
**Chroococcales**									
1	Chroococcales	CCNP 1115							
2		CCNP 1118							
3	*Cyanobacterium* sp.	CCNP 1105							
4	*Microcystis aeruginosa*	CCNP 1101							
5		CCNP 1102							
6	*Microcystis* sp.	CCNP 1106	MIC_SP_						
**Synechococcales**									
7	*Synechocystis salina*	CCNP 1104							
8	*Synechocystis* sp.	CCNP 1108							
9	*Pseudanabaena galeata*	CCNP 1313							
10	*Pseudanabaena* sp.	CCNP 1311							
11		CCNP 1312							
12		KUCC C3			MIC_SP_			MIC_MS_	
13	Pseudanabaenaceae	KUCC C4			MIC_SP_			MIC_MS_	
**Leptolyngbyales**									
14	*Leptolyngbya* sp.	CCNP 1301							
15		CCNP 1302							
16		CCNP 1308							
**Oscillatoriales**									
17	*Phormidium* sp.	CCNP 1317							
18	*Limnoraphis* sp.	CCNP 1314							
19		CCNP 1315							
20		CCNP 1316							
21		CCNP 1324							
22		CCNP 1327							
23		CCNP 1328							
**Spirulinales**									
24	Spirulinales	06S082							
25		CCNP 1310							
**Nostocales**									
26	*Anabaena cylindrica*	CCNP 1405	MIC_MS_		MIC_SP_				
27	*Anabaena* sp.	CCNP 1406	MIC_MS_		MIC_SP_				
28		CCNP 1407		MIC_SP_					
29		CCNP 1416		MIC_SP_					
30		CCNP 1417							
31		CCNP 1419							
32	*Aphanizomenon* sp.	KUCC C1	MIC_MS_						
33		KUCC C2							
34	*Nodularia spumigena*	CCNP 1401							
35		CCNP 1403							
36		LIT 31							
37		CCNP 1430							
38		CCNP 1440							
39	*Nostoc edaphicum*	CCNP 1411							
40	*Nostoc* sp.	CCNP 1420							
41		CCNP 1421							
42		CCNP 1438							
43		CCNP 1445							
44		CCNP 1447							

Yellow shading indicates lack of pigment (violacein) production in the *C. violaceum* ATCC 12472 assay. For *S. pyogenes* ATCC 12344 and *M. smegmatis* ATCC 14468 strains, Minimum Inhibitory Concentration (MIC) values are shown and denoted as MIC_SP_ and MIC_MS_, respectively.

**Table 2:** Antibacterial activity of 21 fractions from the KUCC C1 extract tested against *C. violaceum* ATCC 12472.

Fraction No.	Fraction Concentration [μg/mL]								
	≥8000	4000	2000	1000	500	250	125	63	32
1									
2									
3									
4									
5									
6									
7									
8	-								
9	-	G	G						
10	-	G							
11	-	G	G	G					
12	-	-	-	G	G				
13	-	-	-	-					
14	G	G	G	G	G	G	G	G	
15									
16									
17									
18	-	-	G						
19	-								
20									
21									

Yellow shading indicates antibacterial activity. “-“ denotes a bactericidal effect, whereas “G” indicates bacterial growth without violacein production.

## Data Availability

The data supporting the conclusions of this study are included in the main article and the Supplementary Materials. Raw data are available from the corresponding authors upon reasonable request.

## Supplementary Materials

The following supporting information can be downloaded at: https://www.mdpi.com/article/10.3390/antibiotics15040371/s1, Table S1: Cyanobacterial strains used in the study; Table S2: Antibacterial activity of 44 cyanobacterial extracts against *Streptococcus pyogenes* ATCC 12344, expressed as percentage of bacterial growth relative to the untreated control (100%); Table S3: Antibacterial activity of 44 cyanobacterial extracts against *Mycobacterium smegmatis* ATCC 14468, expressed as percentage of bacterial growth relative to the untreated control (100%); Table S4: Elution times (start and end) and biological activity of 52 fractions obtained from the KUCC C1 crude extract using flash chromatography against *C. violaceum* ATCC 12472. (N—no effect, blue—minimal concentration inhibiting violacein production, red—MBC, M—missing sample); Table S5: OD_585_ values for assays with *C. violaceum* ATCC 12472 exposed to 21 fractions obtained by preparative chromatography (black values—wells with visible turbidity and violet coloration; blue values—turbidity without pigment; red values—wells with no observed turbidity); Table S6: Detected ion signals corresponding to identified diacylglycerols (DAGs), galactosyldiacylglycerols (MGDGs and DGDGs), and their analogs in 21 tested fractions of the KUCC C1 extract.
