# Supplementary material for: Screening of 44 Baltic Sea Cyanobacterial Strains for Antibacterial and Quorum Sensing Inhibitory Potential: Selection of Promising Candidates
Source: Antibiotics (Basel). 2026 Apr 3;15(4):371. doi: 10.3390/antibiotics15040371 (PMC13113589; doi:10.3390/antibiotics15040371)
Supplement: Supplementary file 1 [file antibiotics-15-00371-s001.zip › antibiotics-4207235-supplementary.pdf]

SUPPLEMENTARY MATERIALS

Table S1 Cyanobacterial strains used in the study.
*strains originally isolated for the Klaipeda University Culture Collection, ** strain acquired from Åbo Akademi University in Turku (Finland), *** strain provided by Dr. J. Koreivienė from the Nature Research Centre in Vilnius.

No.	Strain code	Taxon	Isolation place	Isolation year	Harvesting method
**Chroococcales**					
1	CCNP 1115	Chroococcales	Gulf of Gdańsk	2014	Centrifugation
2	CCNP 1118	Chroococcales	Gulf of Gdańsk	2014	Centrifugation
3	CCNP 1105	*Cyanobacterium* sp.	Vistula Lagoon	2010	Centrifugation
4	CCNP 1101	*Microcystis aeruginosa*	Gulf of Gdańsk	2005	Centrifugation
5	CCNP 1102	*Microcystis aeruginosa*	Gulf of Gdańsk	2005	Centrifugation
6	CCNP 1106	*Microcystis viridis*	Gulf of Gdańsk	2006	Centrifugation
**Synechococcales**					
7	CCNP 1104	*Synechocystis salina*	Gulf of Gdańsk	2006	Centrifugation
8	CCNP 1108	*Synechocystis* sp.	Gulf of Gdańsk	2011	Centrifugation
9	CCNP 1313	*Pseudanabaena galeata*	Gulf of Gdańsk	2010	Centrifugation
10	CCNP 1311	*Pseudanabaena* sp.	Gulf of Gdańsk	2009	Centrifugation
11	CCNP 1312	*Pseudanabaena* sp.	Gulf of Gdańsk	2009	Centrifugation
12	KUCC C3*	*Pseudanabaena* sp.	Curonian Lagoon	2020	Centrifugation
13	KUCC C4*	Pseudanabaenaceae	Curonian Lagoon	2020	Centrifugation
**Leptolyngbyales**					
14	CCNP 1301	*Leptolyngbya* sp.	Gulf of Gdańsk	2004	Centrifugation
15	CCNP 1302	*Leptolyngbya* sp.	Puck Bay	2005	Centrifugation
16	CCNP 1308	*Leptolyngbya* sp.	Gulf of Gdańsk	2009	Centrifugation
**Oscillatoriales**					
17	CCNP 1317	*Phormidium* sp.	Gulf of Gdańsk	2014	Centrifugation
18	CCNP 1314	*Limnoraphis* sp.	Gulf of Gdańsk	2014	Cell strainer
19	CCNP 1315	*Limnoraphis* sp.	Gulf of Gdańsk	2014	Cell strainer
20	CCNP 1316	*Limnoraphis* sp.	Gulf of Gdańsk	2014	Cell strainer
21	CCNP 1324	*Limnoraphis* sp.	Gulf of Gdańsk	2012	Cell strainer
22	CCNP 1327	*Limnoraphis* sp.	Gulf of Gdańsk	2020	Cell strainer
23	CCNP 1328	*Limnoraphis* sp.	Gulf of Gdańsk	2020	Cell strainer
**Spirulinales**					
24	06S082**	Spirulinales	Finnish coast	2009	Cell strainer
25	CCNP 1310	Spirulinales	Puck Bay	2009	Cell strainer
**Nostocales**					
26	CCNP 1405	*Anabaena cylindrica*	Gulf of Gdańsk	2005	Centrifugation
27	CCNP 1406	*Anabaena* sp.	Gulf of Gdańsk	2005	Centrifugation
28	CCNP 1407	*Anabaena* sp.	Gulf of Gdańsk	2005	Centrifugation
29	CCNP 1416	*Anabaena* sp.	Gulf of Gdańsk	2011	Centrifugation
30	CCNP 1417	*Anabaena* sp.	Gulf of Gdańsk	2010	Centrifugation
31	CCNP 1419	*Anabaena* sp.	Puck Bay	2012	Centrifugation
32	KUCC C1*	*Aphanizomenon* sp.	Curonian Lagoon	2018	Centrifugation
33	KUCC C2*	*Aphanizomenon* sp.	Curonian Lagoon	2018	Centrifugation
34	CCNP 1401	*Nodularia spumigena*	Gulf of Gdańsk	2003	Centrifugation
35	CCNP 1403	*Nodularia spumigena*	Gulf of Gdańsk	2009	Centrifugation
36	LIT 31***	*Nodularia spumigena*	Gulf of Gdańsk	2016	Centrifugation
37	CCNP 1430	*Nodularia spumigena*	Gulf of Gdańsk	2012	Centrifugation
38	CCNP 1440	*Nodularia spumigena*	Gulf of Gdańsk	2013	Centrifugation
39	CCNP 1411	*Nostoc edaphicum*	Gulf of Gdańsk	2010	Centrifugation
40	CCNP 1420	*Nostoc* sp.	Gulf of Gdańsk	2012	Centrifugation
41	CCNP 1421	*Nostoc* sp.	Gulf of Gdańsk	2012	Centrifugation
42	CCNP 1438	*Nostoc* sp.	Gulf of Gdańsk	2012	Centrifugation
43	CCNP 1445	*Nostoc* sp.	Gulf of Gdańsk	2014	Centrifugation
44	CCNP 1447	*Nostoc* sp.	Gulf of Gdańsk	2014	Centrifugation

Table S2 Antibacterial activity of 44 cyanobacterial extracts against *Streptococcus pyogenes* ATCC 12344, expressed as percentage of bacterial growth relative to the untreated control (100%).

		Extract concentration [µg/mL]								
		1000	500	250	125	63	32	16	8	4
**Chroococcales**										
1	CCNP 1115	109±7	101±1	100±3	100±2	104±5	102±3	99±2	100±1	100±1
2	CCNP 1118	102±1	105±6	101±1	99±5	97±4	101±1	100±3	101±2	100
3	CCNP 1105	143±10	128±3	102±3	100±3	101±2	99±3	103±2	101±1	100±2
4	CCNP 1101	93±4	100±1	104±5	100±2	100±2	100±2	100±1	98±6	99±2
5	CCNP 1102	83±6	97±6	103±4	102±1	100±1	102±3	100±3	101	101±1
6	CCNP 1106	0	20±1	84±5	95±4	94±3	96±5	94±3	94±10	99±6
**Synechococcales**										
7	CCNP 1104	125±4	108±6	104±3	101±3	103±2	100±1	99±2	103	99±2
8	CCNP 1108	131±3	106±5	104±1	102±3	102±2	101±2	101±1	99±2	101±3
9	CCNP 1313	138±8	118±4	102±3	101±2	102±3	101±1	99±2	101±1	102±1
10	CCNP 1311	92±2	101±3	111±7	97±3	103±5	102±2	97±3	98±3	95±6
11	CCNP 1312	109±4	98±8	100	103±7	107±9	107±7	113±6	106±5	104±5
12	KUCC C3	0	0	0	41±4	93±6	101±3	102±3	102±3	99±2
13	KUCC C4	0	0	0	60±2	113±16	113±18	108±15	111±16	115±15
**Leptolyngbyales**										
14	CCNP 1301	129±3	137±7	131±8	128±7	117±10	109±9	104±10	110±6	102±7
15	CCNP 1302	145±5	145±5	120±10	101±2	100±1	102±3	102±3	100±1	100±1
16	CCNP 1308	118±3	114±8	120±9	106±1	101±2	98±4	97±6	100±2	97±6
**Oscillatoriales**										
17	CCNP 1317	119±2	116±4	112±3	109±4	103±2	99±2	105±7	103±5	107±5
18	CCNP 1314	126±4	126±9	115±7	103±3	99±2	94±7	100±3	98±4	99±2
19	CCNP 1315	132±3	150±7	149±5	132±4	124±6	111±10	104±1	101±3	103±3
20	CCNP 1316	131±4	115±5	101±2	100±3	102±4	102±3	99±2	103±2	96±6
21	CCNP 1324	145±5	126±3	122±5	117±6	111±2	107±3	105±5	98±4	100±1
22	CCNP 1327	156±1	139±6	124±5	118±3	106±5	101±2	105±6	101±2	105±6
23	CCNP 1328	149±5	128±6	120±5	107±4	106±6	102±2	107±3	100±1	104±2
**Spirulinales**										
24	06S082	144±12	143±11	138±3	135±10	122±2	121±16	116±9	109±2	103±3
25	CCNP 1310	130±3	144±3	125±5	118±6	109±8	104±5	103±1	101±1	101±2
**Nostocales**										
26	CCNP 1405	0	0	0	80±2	101±2	106±4	109±5	104±8	108±7
27	CCNP 1406	0	0	0	75±11	101±1	101±2	99±4	99±2	97±3
28	CCNP 1407	0	0	94±6	101±2	100±1	101±3	101±2	99±3	101±2
29	CCNP 1416	0	0	98±6	100±2	100±3	99±1	99±2	100±1	100±2
30	CCNP 1417	91±2	94±5	98±4	100±5	100±3	102±4	104±4	101±2	102±2
31	CCNP 1419	102±3	101±2	104±1	103±2	103±3	99±3	102±3	100±1	101±2
32	KUCC C1	114±6	113±3	101±2	101±3	101±3	100±4	104±4	99±3	103±4
33	KUCC C2	101±2	108±8	102±2	101±2	102±4	101±2	100±2	100	102±2
34	CCNP 1401	127±4	107±4	107±6	101±3	102±4	95±6	99±3	99±2	99±2
35	CCNP 1403	110±5	111±3	101±3	104±3	100±1	100±1	92±6	99±3	102±3
36	LIT 31	146±6	135±11	127±10	133±14	124±19	118±7	109±11	107±10	103±3
37	CCNP 1430	130±3	129±6	119±4	114±7	102±2	102±3	103±3	101±1	100±3
38	CCNP 1440	109±3	101±4	99±4	100±4	100±4	97±2	95±8	97±5	99±2
39	CCNP 1411	101±5	99±2	98±5	100±1	98±5	101±2	99±2	101±2	100±1
40	CCNP 1420	119±4	102±3	111±8	104±4	10±8	107±6	101±2	102±3	107±11
41	CCNP 1421	116±10	102±3	103±3	104±3	97±3	96±3	100±3	97±3	99±2
42	CCNP 1438	85±9	109±9	103±4	103±4	103±2	98±5	102±2	101±1	100±4
43	CCNP 1445	126±4	111±3	105±2	104±4	103±1	98±4	104±5	101±2	99±3
44	CCNP 1447	114±6	104±2	101±1	103±1	100	100±1	99±3	98±4	100±1

Table S3 Antibacterial activity of 44 cyanobacterial extracts against *Mycobacterium smegmatis* ATCC 14468, expressed as percentage of bacterial growth relative to the untreated control (100%).

		Extract concentration [µg/mL]								
		1000	500	250	125	63	32	16	8	4
**Chroococcales**										
1	CCNP 1115	130±5	131±10	111±10	101±3	104±3	101±3	130±5	99±1	99±1
2	CCNP 1118	102±2	101±2	101±2	105±5	99±4	100±2	99±1	101±2	100±5
3	CCNP 1105	99±3	105±6	101±3	101±2	99±1	100±1	103±3	103±6	101±2
4	CCNP 1101	103±5	104±4	98±8	101±2	96±6	101±2	103±3	101±2	101±1
5	CCNP 1102	102±1	101±1	104±4	105±5	102±1	104±4	97±7	101±2	98±8
6	CCNP 1106	86±5	73±4	102±1	102±1	98±3	100±1	101±1	101±2	100±1
**Synechococcales**										
7	CCNP 1104	105±4	102±3	101±2	100±2	101±1	101±2	100±2	100±1	100±1
8	CCNP 1108	102±1	102±1	101±2	100	101±2	101±1	102±1	104±6	99±1
9	CCNP 1313	136±4	130±5	105±5	103±3	101±2	102±3	100	101±1	100±1
10	CCNP 1311	102±1	102±2	102±1	100±1	101±1	102±1	101±2	101±1	100±1
11	CCNP 1312	105±5	102±1	103±2	100±1	100±1	105±5	100±2	102±2	100±1
12	KUCC C3	0	0	0	0	0	0	100±2	102±2	100±1
13	KUCC C4	0	0	0	0	0	0	98±2	99±1	100±1
**Leptolyngbyales**										
14	CCNP 1301	127±5	126±4	126±4	106±6	101±2	101±1	101±2	101±2	101±2
15	CCNP 1302	102±3	102±3	100±1	101±1	99±2	100±1	100±1	99±2	99±2
16	CCNP 1308	104±2	99±2	103±3	100±1	100±1	102±2	100±1	101±2	101±2
**Oscillatoriales**										
17	CCNP 1317	101±2	100±2	102±2	101±2	102±2	102±1	101±1	102±2	99±2
18	CCNP 1314	100±1	101±2	96±1	100±2	100	101±1	101±2	100	102±2
19	CCNP 1315	140±5	144±7	121±5	104±3	104±7	102±1	101±2	101±2	100±1
20	CCNP 1316	100±1	101±2	101±2	102±3	103±2	100±1	99±1	102±2	100±1
21	CCNP 1324	103±2	102±2	102±2	101±2	100±1	100±1	100±1	100±1	100±2
22	CCNP 1327	101±3	101±2	100±2	101±2	103±1	101±1	101±2	101±2	101±3
23	CCNP 1328	101±2	100	101±2	101±1	100±2	105±5	101±1	100	102±2
**Spirulinales**										
24	06S082	101±3	104±6	101±1	101±2	112±13	100±1	100±1	100±1	99±2
25	CCNP 1310	105±4	104±5	102±1	101±1	107±6	106±4	101±2	100±1	99±1
**Nostocales**										
26	CCNP 1405	0	57±3	74±7	77±3	101±1	100±1	99±2	102±1	100±1
27	CCNP 1406	0	68±2	67±3	64±3	102±1	100±1	103±4	98±8	101±2
28	CCNP 1407	63±8	64±7	81±7	100±1	100±1	102±3	100±2	97±7	100±1
29	CCNP 1416	68±8	59±4	91±7	101±1	102±2	101±2	100±1	101±1	101±2
30	CCNP 1417	101±2	100±1	100±1	101±2	100±1	99±1	99±2	99±1	101±2
31	CCNP 1419	99±1	99±3	100±2	101±2	101±2	102±1	102±1	101±3	100±1
32	KUCC C1	0	81±1	100±1	100±1	101±3	100±1	102±3	100±1	101±2
33	KUCC C2	99±2	101±2	101±2	101±2	100±1	101±2	101±2	99±2	101±1
34	CCNP 1401	128±4	127±6	103±1	101±2	104±5	102±3	102±2	100±2	100±1
35	CCNP 1403	102±1	103±3	103±2	101±2	101±1	98±3	99±1	101±1	100±3
36	LIT 31	100±1	99±2	101±1	100	100±1	97±2	101±2	103±3	101±2
37	CCNP 1430	100±1	102±3	102±2	103±2	107±3	103±3	102±3	102±2	101±2
38	CCNP 1440	99±2	101±2	102±2	98±3	99±1	102±2	102±2	100±1	100±1
39	CCNP 1411	89±1	99±1	99±2	97±2	101±2	100±2	99±2	101±1	102±2
40	CCNP 1420	89±1	99±1	99±2	97±2	101±1	99±3	96±4	100±1	100±1
41	CCNP 1421	99±2	86±6	100±2	100±1	99±2	101±2	100±1	100±1	102±2
42	CCNP 1438	107±4	101±1	102±3	100±1	102±1	101±2	98±2	100±2	103±2
43	CCNP 1445	110±5	101±2	99±3	103±1	104±5	102±2	99±3	102±3	100±1
44	CCNP 1447	102±4	99±2	99±2	101±1	101±1	102±2	99±3	102±2	99±2

Table S4 Elution times (start and end) and biological activity of 52 fractions obtained from the KUCC C1 crude extract using flash chromatography against *C. violaceum* ATCC 12472 (N - no effect, blue - minimal concentration inhibiting violacein production, red - MBC, M - missing sample).

Fraction no.	Start time	End time	Methanol concentration [%]	Observed effect
1	0.330	2.330		N
2	2.340	4.350		1000
3	4.360	6.360	20	N
4	6.370	8.370		N
5	8.390	10.390		N
6	10.400	12.410		N
7	12.420	14.420		N
8	14.430	16.430		1000
9	16.450	18.450	40	500
10	18.460	20.470		250
11	20.480	22.480		250
12	22.490	24.490		500
13	24.510	26.510		500
14	26.520	28.530		M
15	28.540	30.540		250
16	30.550	32.550	60	63
17	32.590	34.600		63
18	34.610	36.610		125
19	36.620	38.630		125
20	38.640	40.640		1000
21	40.660	42.660		1000
22	42.670	44.680		1000
23	44.690	46.690		M
24	46.700	48.700		250
25	48.720	50.730	80	500
26	50.740	52.740		63
27	52.750	54.750		63
28	54.760	56.770		250
29	56.780	58.790		250
30	58.800	60.800		250
31	60.810	62.810		250
32	62.820	64.820		250
33	64.860	66.870		32
34	66.880	68.880		16
35	68.890	70.890		125
36	70.900	72.910		N
37	72.920	74.930		N
38	74.940	76.940		N
39	76.950	78.950		N
40	78.960	80.970		N
41	80.980	82.990	100	N
42	83.000	85.000		N
43	85.010	87.010		N
44	87.020	89.030		N
45	89.040	91.050		N
46	91.060	93.060		N
47	93.070	95.070		M
48	95.080	97.090		N
49	97.120	99.130		N
50	99.140	101.140		N
51	101.150	103.160		N
52	103.170	104.030		M

Table S5 OD_{585} values for assays with *C. violaceum* ATCC 12472 exposed to 21 fractions obtained by preparative chromatography (black values – wells with visible turbidity and violet coloration; blue values – turbidity without pigment; red values – wells with no observed turbidity).

Fraction no.	Fraction concentration [µg/mL]									
	≥8000	4000	2000	1000	500	250	125	63	32	Ringer solution
**1**	0,738	0,726	0,727	0,735	0,740	0,740	0,725	0,741	0,729	0,100
	0,741	0,730	0,725	0,726	0,742	0,733	0,730	0,733	0,733	0,099
**2**	0,729	0,728	0,732	0,733	0,744	0,743	0,740	0,740	0,746	0,104
	0,736	0,745	0,740	0,743	0,729	0,745	0,738	0,744	0,730	0,102
**3**	0,730	0,728	0,730	0,741	0,732	0,740	0,735	0,732	0,744	0,101
	0,741	0,733	0,729	0,744	0,746	0,729	0,740	0,733	0,729	0,103
**4**	0,740	0,742	0,742	0,737	0,740	0,733	0,732	0,731	0,735	0,099
	0,729	0,730	0,740	0,739	0,735	0,731	0,740	0,743	0,736	0,100
**5**	0,735	0,733	0,740	0,732	0,730	0,738	0,730	0,741	0,745	0,101
	0,742	0,733	0,733	0,740	0,745	0,740	0,740	0,728	0,729	0,103
**6**	0,741	0,729	0,736	0,731	0,739	0,738	0,742	0,737	0,734	0,105
	0,740	0,731	0,740	0,737	0,740	0,741	0,740	0,739	0,733	0,102
**7**	0,733	0,736	0,737	0,740	0,740	0,729	0,740	0,733	0,740	0,100
	0,725	0,730	0,735	0,726	0,741	0,741	0,737	0,737	0,729	0,099
**8**	0,150	0,720	0,735	0,732	0,736	0,740	0,751	0,741	0,732	0,103
	0,139	0,735	0,740	0,735	0,742	0,749	0,740	0,743	0,746	0,098
**9**	0,145	0,359	0,368	0,750	0,729	0,736	0,731	0,745	0,738	0,101
	0,139	0,360	0,370	0,733	0,731	0,735	0,736	0,745	0,742	0,102
**10**	0,151	0,355	0,720	0,700	0,720	0,746	0,741	0,733	0,733	0,100
	0,149	0,360	0,703	0,712	0,720	0,735	0,736	0,759	0,762	0,105
**11**	0,138	0,359	0,368	0,359	0,725	0,734	0,745	0,753	0,733	0,100
	0,144	0,366	0,348	0,361	0,735	0,739	0,752	0,751	0,737	0,103
**12**	0,138	0,139	0,135	0,350	0,355	0,744	0,750	0,763	0,740	0,099
	0,138	0,149	0,141	0,361	0,348	0,731	0,741	0,711	0,704	0,102
**13**	0,145	0,139	0,137	0,146	0,777	0,699	0,733	0,669	0,735	0,101
	0,150	0,151	0,142	0,133	0,750	0,723	0,787	0,727	0,744	0,100
**14**	0,350	0,344	0,366	0,352	0,341	0,347	0,391	0,355	0,718	0,105
	0,356	0,339	0,369	0,346	0,360	0,311	0,365	0,348	0,700	0,097
**15**	0,740	0,734	0,721	0,735	0,766	0,755	0,758	0,758	0,745	0,100
	0,745	0,730	0,750	0,752	0,745	0,741	0,744	0,744	0,750	0,101
**16**	0,737	0,737	0,732	0,746	0,750	0,738	0,742	0,744	0,740	0,099
	0,743	0,717	0,750	0,745	0,740	0,745	0,742	0,737	0,733	0,100
**17**	0,726	0,763	0,750	0,762	0,733	0,731	0,740	0,797	0,740	0,102
	0,733	0,743	0,749	0,739	0,741	0,741	0,737	0,752	0,731	0,100
**18**	0,145	0,156	0,323	0,757	0,731	0,740	0,751	0,731	0,746	0,101
	0,138	0,130	0,370	0,739	0,743	0,749	0,770	0,717	0,744	0,100
**19**	0,140	0,751	0,747	0,717	0,733	0,735	0,751	0,756	0,750	0,103
	0,145	0,747	0,719	0,766	0,733	0,744	0,758	0,761	0,740	0,100
**20**	0,742	0,732	0,735	0,740	0,730	0,741	0,745	0,730	0,750	0,099
	0,740	0,740	0,740	0,743	0,741	0,728	0,729	0,740	0,740	0,104
**21**	0,737	0,739	0,736	0,745	0,742	0,737	0,735	0,742	0,742	0,100
	0,733	0,738	0,741	0,729	0,740	0,739	0,733	0,741	0,744	0,100

Table S6 Detected ion signals corresponding to identified diacylglycerols (DAGs), galactosyldiacylglycerols (MGDGs and DGDGs), and their analogues in 21 tested fractions of the KUCC C1 extract.

RT	*m/z*	Fraction number									
		1-7	8	9	10	11	12	13	14	15	16-21
12,43	891,61				5,14E+05		5,40E+05	7,09E+05	1,68E+05		
11,82	529,37					2,86E+05			1,85E+05		
12,42	526,41				2,02E+05		2,47E+05	6,61E+05	3,48E+05		
12,65	526,42							1,30E+06	4,03E+05		
12,32	703,39					3,66E+06			4,21E+05		
12,35	636,45							7,69E+05	4,57E+05		
12,54	704,49					2,16E+06			4,77E+05		
13,57	657,37								5,75E+05		
13,12	528,46				2,38E+05			6,25E+05	7,04E+05		
11,22	536,40					6,42E+05			8,32E+05		
12,70	539,12								1,07E+06		
13,70	623,39							2,75E+05	1,66E+06		
13,35	627,20								2,30E+06		
11,66	699,42					1,07E+06		1,58E+05			
12,60	943,78							1,70E+05			
11,67	566,37			4,93E+05	6,04E+05			2,25E+05			
12,36	541,42		1,24E+05					2,36E+05			
11,09	571,40				2,06E+05		1,15E+05	2,63E+05			
11,96	633,40				9,01E+05			4,88E+05			
12,65	890,58					7,79E+05		5,39E+05			
11,86	671,45				3,16E+05	1,01E+06		5,79E+05			
11,93	649,37			4,76E+05	9,72E+05	4,29E+05		5,92E+05			
13,01	548,47							6,57E+05			
12,80	989,63					1,68E+05		7,55E+05			
12,13	885,65							8,69E+05			
12,24	697,44				1,40E+06			8,73E+05			
12,89	715,51					2,23E+05		8,96E+05			
12,87	642,32							9,04E+05			
12,32	879,61				2,93E+05	3,01E+05	3,25E+05	1,24E+06			
2,05	556,35						1,30E+05				
11,95	698,51					2,80E+06	1,32E+05				
11,84	734,49				6,55E+05		1,35E+05				
11,38	640,44		1,68E+05				1,37E+05				
11,70	1001,60				3,64E+05	3,80E+05	1,50E+05				
11,85	1044,79						1,52E+05				
10,89	569,37					4,05E+05	1,52E+05				
11,96	722,47		1,13E+05	8,44E+05	4,18E+05	1,06E+06	1,66E+05				
10,15	589,30						1,67E+05				

Table S6 continued

RT	m/z	1-7	8	9	10	11	12	13	14	15	16-21
11,84	504,39				7,33E+05	1,33E+06	1,85E+05				
11,70	996,65				3,82E+05	2,23E+05	1,92E+05				
10,92	546,35						1,95E+05				
11,82	694,39			2,03E+06	1,03E+06	5,17E+05	2,12E+05				
11,74	700,05						2,33E+05				
11,51	564,93						2,43E+05				
11,32	564,39					3,04E+05	2,52E+05				
11,72	875,49					1,18E+06	2,55E+05				
11,70	889,76			1,84E+05		1,06E+05	2,88E+05				
12,10	727,39			1,18E+06	1,46E+06	1,48E+06	2,91E+05				
10,32	543,36					5,74E+05	3,66E+05				
11,88	1029,62				2,67E+05	1,22E+05	3,74E+05				
11,17	640,41						3,85E+05				
12,15	729,43		4,02E+05		9,86E+05		4,27E+05				
11,88	670,39					1,62E+06	4,44E+05				
13,78	512,91						6,07E+05				
12,48	638,45						6,35E+05				
11,79	701,36		1,35E+05		2,59E+06		8,35E+05				
11,93	839,53						1,11E+06				
12,23	506,36						1,76E+06				
13,10	729,45						2,06E+06				
6,15	530,33					7,05E+04					
2,23	628,93					7,82E+04					
2,25	629,28					1,10E+05					
11,40	732,36					1,20E+05					
11,22	575,40				2,68E+05	1,29E+05					
14,84	736,53					1,32E+05					
11,69	631,39			9,31E+05		1,43E+05					
9,80	615,33					1,44E+05					
11,05	523,30		3,20E+05			2,00E+05					
11,62	539,37		2,89E+05			2,08E+05					
11,54	525,33		4,00E+05			2,45E+05					
9,92	555,29					2,58E+05					
2,34	601,21			1,29E+05	1,48E+05	3,10E+05					
9,66	601,32					3,19E+05					
10,21	542,35					3,61E+05					
11,03	553,35					4,71E+05					
9,42	573,29					4,92E+05					
10,64	525,32					5,87E+05					
9,57	559,33					6,26E+05					
9,92	587,34					6,40E+05					

Table S6 continued

RT	m/z	1-7	8	9	10	11	12	13	14	15	16-21
11,70	925,57					9,11E+05					
10,21	541,35					9,48E+05					
11,70	924,53					1,40E+06					
12,51	565,33					1,90E+06					
12,48	731,37					2,27E+06					
10,79	515,30				1,15E+05						
11,70	830,52		1,70E+05	4,73E+05	1,22E+05						
10,88	687,45				1,26E+05						
10,78	689,46				1,55E+05						
11,58	666,36			2,20E+06	1,67E+05						
10,91	705,38				1,76E+05						
9,90	723,42				1,87E+05						
10,58	545,30				2,20E+05						
11,99	865,51			2,53E+05	2,36E+05						
10,45	705,37				2,45E+05						
11,70	856,57			3,95E+05	2,71E+05						
11,52	861,55				3,26E+05						
12,99	502,35				3,57E+05						
11,77	503,37				3,62E+05						
11,63	832,54				5,89E+05						
11,63	837,47				6,74E+05						
11,71	674,34				2,00E+06						
11,68	836			8,01E+04							
11,79	702,42			3,65E+05							
11,70	835,46		2,42E+05	3,79E+05							
11,98	695,38			1,28E+06							
11,76	667,40			1,33E+06							
11,57	997,56		1,05E+05								
11,96	756,48		1,19E+05								
11,22	679,40		1,42E+05								
11,57	629,38		1,46E+05								
12,24	541,36		1,88E+05								
11,49	833,50		2,00E+05								
11,73	728,44		7,16E+05								
